# HADHA Regulates Respiratory Complex Assembly and Couples FAO and OXPHOS

**DOI:** 10.1002/advs.202405147

**Published:** 2024-11-03

**Authors:** Chaoying Qin, Shasha Gong, Ting Liang, Zhenbo Zhang, Jessie Thomas, Janice Deng, Yaguang Liu, Peiqing Hu, Bi Zhu, Shujie Song, Marisol Fernández Ortiz, Yuji Ikeno, Exing Wang, James Lechleiter, Susan T. Weintraub, Yidong Bai

**Affiliations:** ^1^ Department of Cell Systems and Anatomy The University of Texas Health San Antonio San Antonio Texas 78229 USA; ^2^ Xiangya Hospital Central South University Changsha Hunan 410008 China; ^3^ Taizhou Central Hospital (Taizhou University Hospital) Medical School Taizhou University Taizhou Zhejiang 318000 China; ^4^ Barshop Institute of Aging Research and Longevity and Department of Pathology University of Texas Health San Antonio San Antonio Texas 78229 USA; ^5^ Geriatric Research Education and Clinical Center Audie L. Murphy VA Hospital South Texas Veterans Health Care System San Antonio TX 78229 USA; ^6^ Department of Biochemistry and Structural Biology The University of Texas Health San Antonio San Antonio Texas 78229 USA; ^7^ Population Science and Prevention Program, Mays Cancer Center The University of Texas Health San Antonio San Antonio Texas 78229 USA

**Keywords:** fatty acid oxidation (FAO), HADHA, mitochondrial respiratory chain, mitochondrial trifunctional protein (MTP), respiratory complex I

## Abstract

Oxidative phosphorylation (OXPHOS) and fatty acid oxidation (FAO) are key bioenergetics pathways. The machineries for both processes are localized in mitochondria. Secondary OXPHOS defects have been documented in patients with primary FAO deficiencies, and vice versa. However, the underlying mechanisms remain unclear. Intrigued by the observations that regulation of supercomplexes (SCs) assembly in a mouse OXPHOS deficient cell line and its derivatives is associated with the changes in lipid metabolism, a proteomics analysis is carried out and identified mitochondrial trifunctional protein (MTP) subunit alpha (hydroxyacyl‐CoA dehydrogenase trifunctional multienzyme complex subunit alpha, HADHA) as a potential regulatory factor for SCs assembly. HADHA‐Knockdown cells and mouse embryonic fibroblasts (MEFs) derived from HADHA‐Knockout mice displayed both reduced SCs assembly and defective OXPHOS. Stimulation of OXPHOS induced in cell culture by replacing glucose with galactose and of lipid metabolism in mice with a high‐fat diet (HFD) both exhibited increased HADHA expression. HADHA Heterozygous mice fed with HFD showed enhanced steatosis associated with a reduction of SCs assembly and OXPHOS function. The results indicate that HADHA participates in SCs assembly and couples FAO and OXPHOS.

## Graphical abstract

Through a proteomic approach, a key enzyme is identified in fatty acid oxidation, HADHA, which played an important role in respiratory supercomplex assembly. It provides evidence for the coupling of FAO and OXPHOS. This finding explains the secondary defects of one bioenergetics pathway resulting from the primary deficiency of the other. It thus underscores new treatments for some metabolic diseases.

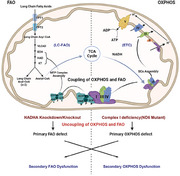

## 1 Introduction

Mitochondria are essential organelles for bioenergetic and metabolic processes.^[^ ^1^ ^]^ The cellular energy currency ATP is mainly produced in mitochondria where fat‐derived fatty acids and carbohydrate‐derived pyruvate are further oxidized through respective metabolic pathways and the products then merge at the tricarboxylic acid cycle (TCA), and ultimately drive OXPHOS.^[^ ^2^ ^]^

OXPHOS is carried out by electron transport chain (ETC) complexes and ATP synthase located at the distinctive regions of the mitochondrial inner membrane.^[^ ^3^, ^4^ ^]^ Following the development of mild detergent and high‐quality native gel electrophoresis, blue native polyacrylamide gel electrophoresis (BN‐PAGE), the organization of ETC complexes has been re‐examined.^[^ ^5^ ^]^ An emerging model for ETC organization termed supercomplexes (SCs), where multiple respiratory complexes assemble together,^[^ ^6^ ^]^ allows for more efficient and effective electron transport and a stabilized respiratory chain.^[^ ^7^, ^8^ ^]^ The arrangement of ETC was shown to be dependent on cristae remodeling, revealing a link between mitochondria morphology and bioenergetic function.^[^ ^9^ ^]^ Accumulating reports have listed several molecules that regulate SCs dynamics including cardiolipin^[^ ^4^, ^10^ ^]^ and COX7RP,^[^ ^11^ ^]^ among others. Not surprisingly, disruption of SCs formation was shown to lead to mitochondrial dysfunction. Reduced SCs stability was also suggested to contribute to the pathogenesis of human diseases like Barth syndrome.^[^ ^12^, ^13^ ^]^ Enhanced supercomplex assembly was suggested to associate with longevity and mitigating age‐related degenerations.^[^ ^14^ ^]^

Mitochondrial fatty acid oxidation involves a repeated sequence of reactions that result in the conversion of fatty acids to acetyl‐CoA. It is carried out by a series of enzymes and transport proteins that successively cleave acetyl‐CoA fragments from fatty acyl‐CoA thioesters until the fatty acyl‐CoA is completely degraded.^[^ ^15^ ^]^ The resulting acetyl‐CoA is further oxidized in the TCA cycle and ultimately delivers high‐energy electrons to the OXPHOS system. Each cycle of oxidation in this pathway is composed of four enzyme activities: acyl‐CoA dehydrogenase (ACAD),^[^ ^16^ ^]^ 2‐enoyl‐CoA hydratase (ECH), 3‐hydroxyacyl‐CoA dehydrogenase (HAD), and 3‐ketothiolase (KT).^[^ ^17^ ^]^ In mammalian mitochondria, the degradation of long‐chain fatty acids is performed by two protein complexes residing on the mitochondrial inner membrane. While the first reaction is carried out by very long chain acyl‐CoA dehydrogenase (VLCAD),^[^ ^18^ ^]^ the next three reactions are performed by a single protein complex, MTP.^[^ ^19^ ^]^ MTP is composed of two subunits: while the α‐subunit contains the activities of ECH and HAD, the β‐subunit bears the remaining KT activity.^[^ ^20^ ^]^ It has been recently shown that while multiple αnβn (n = 2, 3, 4,…) have been identified, the biological unit of MTP is α2β2. The overall structure is a tightly bound homodimer of two β‐subunits at the center with two α‐subunits bounded to each side of the β2 dimer.^[^ ^17^ ^]^ The α2β2 binds on its concave side to the inner membrane of mitochondria.

The majority of human metabolic diseases are correlated with mitochondrial disorders, which are often attributed to dysfunction of the OXPHOS system and have a high prevalence of 1 per 5000 live births.^[^ ^21^, ^22^ ^]^ The main features of OXPHOS disorders are defects in activity and steady‐state levels of the OXPHOS complexes.^[^ ^23^, ^24^ ^]^ Meanwhile, FAO disorders, which can occur in any stage of the FAO pathway, are present as a variety of clinical phenotypes.^[^ ^25^ ^]^ Mutations in both subunits of MTP can result in reduced activity of MTP enzyme activities, and at least 32 mutations in the α‐gene and 30 mutations in the β‐gene have been associated with diseases. Clinical phenotypes of dysfunctional MTP include hypoketotic hypoglycemia^[^ ^21^ ^]^ associated with metabolic acidosis, cardiomyopathy,^[^ ^26^ ^]^ retinopathy,^[^ ^27^ ^]^ and several liver diseases.^[^ ^16^ ^]^ Interestingly, secondary OXPHOS disorders were found in patients who carry mutations in FAO pathways, and secondary FAO defects are also associated with primary OXPHOS dysfunction.^[^ ^28^ ^]^

To investigate the regulation of OXPHOS, we generated a series of cell models with defective or upregulated assembly of respiratory complexes.^[^ ^29^, ^30^, ^31^, ^32^ ^]^ In particular, a cell line 4A, derived from mouse A9 by selection for resistance to a complex I inhibitor rotenone, carries a nearly homoplasmic ND6 frameshift mutation in the mitochondrial DNA (mtDNA), which leads to nearly complete loss of ND6 expression, and loss of complex I and overall OXPHOS activity.^[^ ^29^ ^]^ A 4A derivative cell line, named 4AR6, was isolated for recovery of OXPHOS activity determined by its survival in a galactose medium. 4AR6 cells maintained the absence of ND6 subunit as in 4A cells but regained complex I and overall OXPHOS activity by a compensatory mechanism programmed in the nuclear genome.^[^ ^33^ ^]^

In the present study, we explored the regulated complex I‐containing supercomplex assembly in 4AR6 cells and identified the association of mitochondrial OXPHOS and FAO. Employing molecular, biochemical, proteomics, and physiology approaches in cell and mouse models, we carried out comprehensive studies on the underlying mechanisms of such coupling of bioenergetic pathways and their potential implications in diseases.

## 2 Results

### 2.1 The Regulation of SCs Assembly and Lipid Metabolism in Suppression of Respiratory Dysfunction in a Cell Model with a Complex I Defect

**Figure 1:**
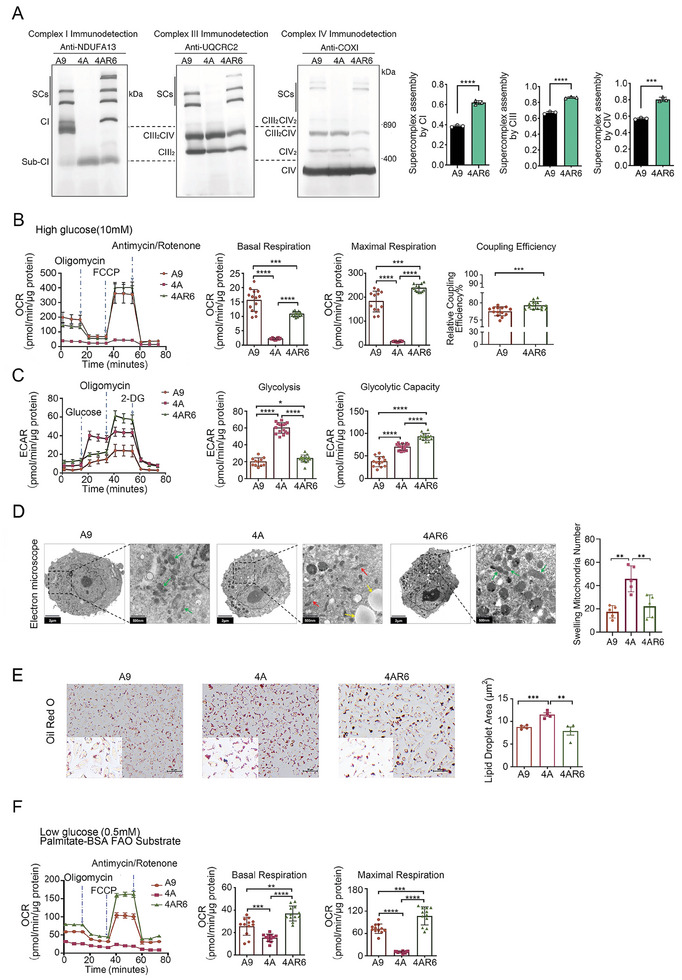
The regulation of SCs assembly and lipid metabolism in suppression of respiratory dysfunction in cell model with complex I defect. A) Evaluation of the Supercomplexes(SCs) assembly rate by BN‐PAGE in A9, 4A, and 4AR6 cells. n = 3 per group. B) Evaluation of the mitochondrial OXPHOS function by mitochondrial oxygen consumption rate (OCR) in A9, 4A, and 4AR6 cells. n = 12‐14. C) Evaluation of the glycolysis function by extracellular acidification rate (ECAR) in A9, 4A, and 4AR6 cells, n = 13‐15. D) EM analysis of mitochondrial morphology of A9, 4A, and 4AR6 cells. The mitochondria swelling in A9, 4A, and 4AR6 cells is quantified and shown by bar graph. n = 3‐5. Scale bars, 2 µm and 500 nm. E) Representative images of Oil red O staining of Lipid in A9, 4A, and 4AR6 cells, and quantitative analysis of lipid droplets area. n = 4 per group. Scale bars, 50 µm. F) Evaluation of the fatty acid oxidation by OCR in A9, 4A, and 4AR6 cells. n = 11‐13. Data are mean ± SEM. *p < 0.05; **p < 0.01;***p < 0.001; ****p < 0.0001. BN‐PAGE: Blue Native Polyacrylamide Gel Electrophoresis.

To investigate the underlying mechanism of OXPHOS restoration in 4AR6 cells, a derivative of a respiratory mutant 4A cell containing ND6 mutation,^[^ ^33^ ^]^ we performed BN‐PAGE to follow the assembly of respiratory machinery. As shown in **Figure** **1**A, mutant 4A was not capable of complex I and complex I‐containing SCs assemblies due to the absence of the ND6 subunit. However, complex I and SCs assembly in 4AR6 cells was restored and even enhanced compared to the 4A parental A9 cells determined by antibodies against complex I, III, and IV subunits. In addition, a higher molecular SC and a shift in individual complex I assembly were detected in 4AR6 cells (Figure 1A).

To determine if the regulated assembled respiratory chain in 4AR6 cells was functional, a series of bioenergetics assays measuring OXPHOS under various conditions were performed in the original A9, mutant 4A, and the mutant suppressor 4AR6 cells. OXPHOS features were determined by measuring oxygen consumption rate (OCR) at baseline and after sequential addition of oligomycin, Carbonyl cyanide‐p‐trifluoromethoxyphenylhydrazone (FCCP), and Antimycin/Rotenone (Figure 1B). As shown in previous studies, basal respiration was extremely low in 4A, and basal respiration of 4AR6 was much improved compared to 4A cells. We also found a significant increase in maximal respiration in 4AR6 measured by the addition of FCCP, which was even higher than that of A9 cells (Figure 1B), consistent with the enhancement observed in complex I and SCs assembly (Figure 1A). The mitochondrial ATP production coupled respiration was determined by calculating the difference of measurements in the absence and presence of oligomycin, which is an ATP synthesis (complex V) inhibitor. Interestingly, as shown in Figure 1B, the OXPHOS coupling efficiency, as determined by the ratio of oligomycin‐sensitive respiration to total basal respiration, was also upregulated in 4AR6 cells.

Glycolysis was determined by measuring the changes in extracellular acidification rate (ECAR) following sequential addition of glucose, oligomycin, and 2‐deoxy‐D‐glucose (2‐DG). Glycolysis was shown to be upregulated in 4A, likely to compensate for the OXPHOS failure (Figure 1C). Meanwhile, 4AR6 had the highest reserved glycolytic capacity, although it mostly relied on OXPHOS for ATP production (Figure 1C).

Interestingly, accompanying the change in OXPHOS function, we also found differences in cell morphologies in 4A and 4AR6 cells. Electron microscopy (EM) was used to observe the morphological features of A9, 4A, and 4AR6 cells. As expected, we found an accumulation of swollen and broken mitochondria in the mutant 4A cells, while mitochondria in 4AR6 were as healthy as those in A9 cells (Figure 1D). Surprisingly, in addition to the changes in mitochondria, a striking feature in 4A cells was the accumulation of lipid droplets, and such accumulation disappeared in 4AR6 cells (Figure 1D). We confirmed the nature and the changes in lipid droplets with Oil Red O tests. As shown in Figure 1E, lipid droplets increased significantly in mutant 4A cells compared with parental wildtype A9 cells. Accompanying the suppression of OXPHOS dysfunction, 4AR6 cells also exhibited repairment in defects in lipid metabolism. These results indicated a change in lipid metabolism concurrent with the alterations in OXPHOS.

While the endoplasmic reticulum (ER) is the main site for lipid synthesis, its degradation mostly happens in mitochondria through FAO. We then used a seahorse Extracellular Flux Analyzer to examine FAO‐dependent respiration. LC‐FAO‐dependent respiration was determined by measuring OCR in a medium with minimal glucose but BSA‐palmitate as a long‐chain fatty acid substrate. While 4A possessed the lowest oxidation capacity in all measurements, 4AR6 showed the highest levels of FAO‐dependent basal and maximal respirations (Figure 1F).

### 2.2 The Upregulation of HADHA in 4AR6 Cells

**Figure 2:**
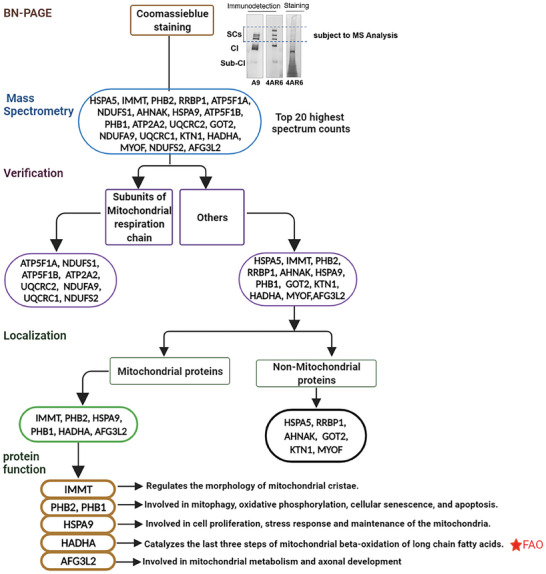
The identification of HADHA as the candidate for coupling of OXPHOS and FAO.

To exclude the possibility that the suppression of respiratory dysfunction was due to reversion at the mtDNA level, we sequenced the whole mitochondrial genomes of 4AR6, with A9 and 4A as controls. Consistent with our previous determination of lack of ND6 protein in 4AR6 cells with mitochondrial protein synthesis and primer‐extension assay at DNA level,^[^ ^33^ ^]^ we found no additional changes in mtDNA from 4AR6 compared with 4A cells (the sequencing results of the ND6 portion are shown in Figure S1, Supporting Information). Based on the observation that complex I and SC bands were shifted in 4AR6 cells (**Figure** **2**), we hypothesized that the restoration of complex I and SCs assembly involved binding of protein factor(s) that facilitated the complex I and supercomplex assembly in the absence of ND6.

To identify the putative supercomplex assembly factor(s), we carried out a proteomics analysis of SCs in 4AR6. As shown in Figure 2, the area on BN‐PAGE that exhibited specific SC assembly in 4AR6 cells, compared with A9, was subjected to mass spectrometry analysis. The top 20 identified proteins with the highest spectrum counts were selected for further consideration. Among these 20 proteins, we found 8 of them were respiratory complex subunits: 3 for complex I, 2 for complex III, and 3 for complex V. With the remaining 12 proteins, we excluded 6 with established localization other than mitochondria. Among the remaining 6 mitochondrial proteins, we focused our attention to hydroxyacyl‐CoA dehydrogenase trifunctional multienzyme complex subunit alpha (HADHA), the a2 subunit of MTP, for its role in lipid metabolism.

**Figure 3:**
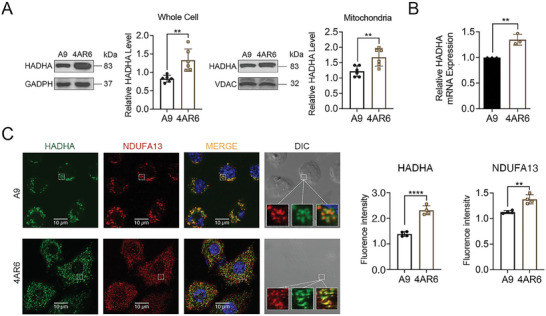
The upregulation of HADHA in 4AR6 cells. A) Western blot of HADHA expression level in A9 and 4AR6 cells. n = 6 per group. B) RT‐PCR analysis of the mRNA expression levels of A9 and 4AR6. A9 was regarded as control. n = 3 per group. C) Representative images of immunofluorescence staining in A9 and 4AR6 cells. n = 4 per group. Data are mean ± SEM. **p < 0.01;***p < 0.001; ****p < 0.0001.

Enhanced expression of HADHA in 4AR6 cells was detected with western blot analysis of protein extracts from whole cells and purified mitochondria of A9 and 4AR6 cells (**Figure** **3**A). RT‐qPCR was also employed to measure the transcription of HADHA (Figure 3B). A significant increase of ≈30% was recorded in all 3 measurements (Figure 3A,B). HADHA expression and localization were further verified with confocal immunofluorescence microscopy with antibodies against HADHA and NDUFA13, a mitochondrial respiratory complex I subunit as the mitochondrial marker. As shown in Figure 3C, HADHA was predominantly localized to mitochondria. The quantitative colocalization analysis was carried out using the Jacop plugin on Fiji software. For A9 cells, Li's intensity correlation co‐efficient (ICQ)^[^ ^34^ ^]^ is 0.39 (in the range of ‐0.50‐0.50), and Mander's Coefficients (Costes, 2004) is M1 (fraction of HADHA overlapping NDUFA13) at 0.62 (in the range 0 to 1.00) and M2 (fraction of NDUFA13 overlapping HADHA) at 0.803 (in the range 0 to 1.00). For 4AR6 cells: Li's ICQ is 0.39; M1 is 0.79 and M2 is 0.82. These results indicate the increased part of HADHA is more likely localized to mitochondria. The fluorescence intensity of HADHA was higher in 4AR6 cells and correlated with the higher level of complex I as detected by an antibody against its subunit NDUFA13 in 4AR6 cells (Figure 3C).

### 2.3 Reduction of HADHA Decreased SCs Assembly and Respiratory Function

**Figure 4:**
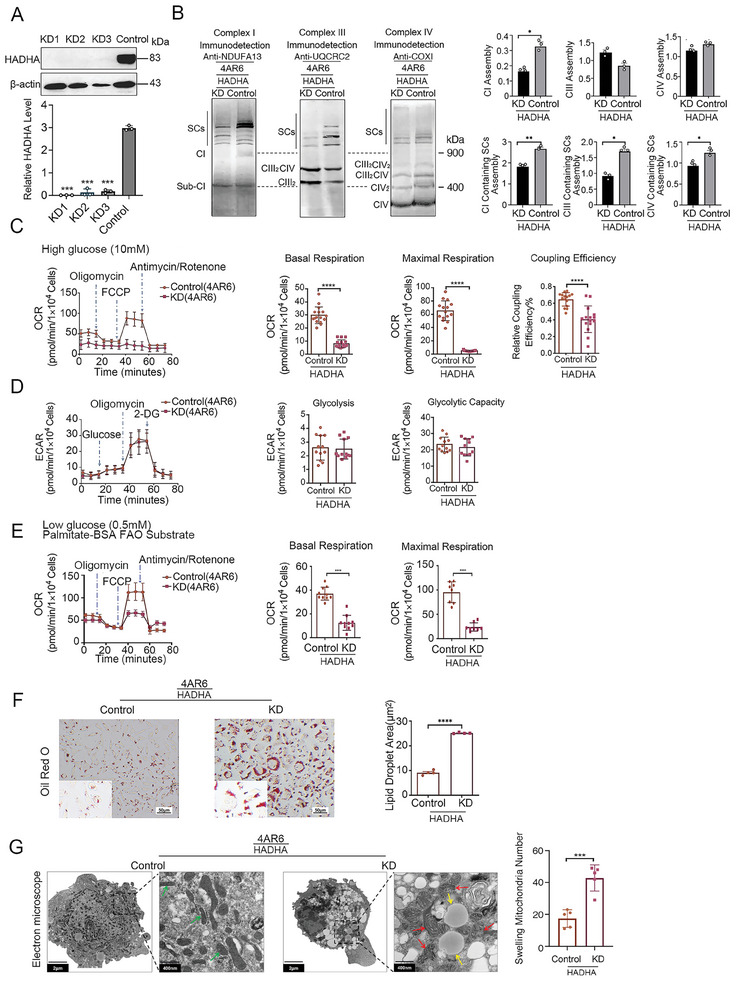
Reduction of HADHA decreased SCs assembly and respiration function. A) Western blot of HADHA expression level in 4AR6‐HADHA‐KD(KD1, KD2, KD3) and 4AR6‐HADHA‐control cells. n = 3 per group. B) Evaluation of the individual complex and supercomplex assembly by BN‐PAGE in 4AR6‐HADHA‐KD3 and 4AR6‐HADHA‐control cells. n = 3 per group. C) Evaluation of the mitochondrial OXPHOS function by OCR in 4AR6‐HADHA‐KD3 and 4AR6‐HADHA‐control cells. n = 12‐14. D) Evaluation of the glycolysis function by ECAR in 4AR6‐HADHA‐KD3 and 4AR6‐HADHA‐control cells. n = 13‐15. E) Evaluation of the fatty acid oxidation by OCR in 4AR6‐HADHA‐KD3 and 4AR6‐HADHA‐control cells. n = 11‐13. F) Representative images of Oil red O staining of Lipid in 4AR6‐HADHA‐KD3 and 4AR6‐HADHA‐control cells, and quantitative analysis of lipid droplets area. n = 4 per group. Scale bars, 50 µm. G) EM analysis of mitochondrial morphology of A9, 4A, and 4AR6 cells. The mitochondria swelling in 4AR6‐HADHA‐KD3 and 4AR6‐HADHA‐control cells is quantified and shown by bar graph. n = 3. Scale bars, 2 µm and 500nm. Data are mean ± SEM. *p < 0.05, **p < 0.01, ***p < 0.001, ****p < 0.0001, ns, nonsignificant. KD: knockdown, EM: electron microscope, SCs: supercomplexes. OCR: oxygen consumption rate, ECAR: extracellular acidification rate. BN‐PAGE: Blue Native Polyacrylamide Gel Electrophoresis.

To confirm the role of HADHA in OXPHOS machinery assembly, we generated 4AR6‐HADHA‐Knockdown(4AR6‐HADHA‐KD) cells by transfecting 4AR6 cells with a construct expressing HADHA shRNA, while the controls were 4AR6 transfected by the same expressing vector with no inserts. Three independent 4AR6‐HADHA‐KD clones were tested for HADHA knockdown efficiency. We confirmed that HADHA expression was largely suppressed in all 3 clones (**Figure** **4**A). We selected KD1 as 4AR6‐HADHA‐KD and 4AR6 transfected with empty vector as control. BN‐PAGE was then performed with digitonin‐treated lysates of 4AR6‐HADHA‐KD clones and control cells. Complex I, III, and IV containing respiratory complexes were detected with specific antibodies (Figure 4B). With the knockdown of HADHA, complex I and SCs assembly decreased significantly, while no obvious effects on complex III and IV were observed (Figure 4B).

Bioenergetic features were then investigated with the Seahorse XF^96^ extracellular flux analyzer on 4AR6‐HADHA‐KD cells and their controls. As shown in Figure 4C, both basal and maximal respirations decreased significantly in 4AR6‐HADHA‐KD cells. In addition, electron transfer and ATP production coupling efficiency, as determined by the ratio of oligomycin sensitive portion to basal respiration, was compromised with the reduction of HADHA level. Meanwhile, both the baseline and the capacity of glycolysis didn't exhibit any obvious changes with the decrease of HADHA (Figure 4D).

To verify the connection between lipid metabolism, FAO in particular, and OXPHOS, we further measured FAO‐dependent respiration rates in 4AR6‐HADHA‐KD cells. As shown in Figure 4E, significant decreases in FAO‐dependent basal and maximal respirations were recorded in 4AR6‐HADHA‐KD cells.

FAO‐associated lipid metabolism dysfunction was confirmed by an oil red O test as more lipid droplets were accumulated in 4AR6‐HADHA‐KD cells (Figure 4F). Electron microscopy (EM) analysis was then applied to investigate morphological features associated with alterations in HADHA expression. While dramatic increases in lipid droplets were observed in 4AR6‐HADHA‐KD cells (Figure 4G), we also found swollen mitochondria, similar to what was observed in OXPHOS deficient 4A cells (Figure 1D).

**Figure 5:**
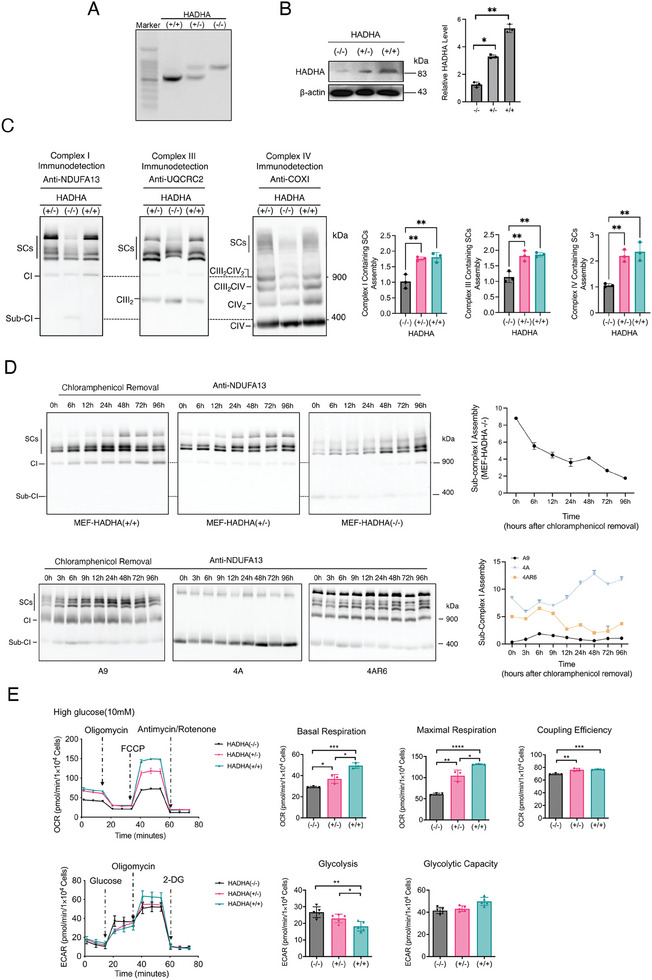
The determination of role of HADHA in respiratory chain assembly and OXPHOS with MEFs with different expression of HADHA. A) verification of mice MEFs’ genotypes by specific PCR. n = 3. B) Western blot of HADHA expression level in WT(HADHA+/+), HADHA‐HET(HADHA±), and HADHA‐HOMO(HADHA‐/‐) MEFs. n = 3 per group. C) Evaluation of the respiratory complexes and supercomplexes assembly by BN‐PAGE in WT, HADHA‐HET, and HADHA‐homo mice MEFs. n = 3 per group. D) Kinetic recovery of respiratory complexes and supercomplexes assembly. Evaluation of the respiratory complexes and supercomplexes assembly by BN‐PAGE in chloramphenicol‐treated WT, HADHA‐HET, and HADHA‐homo MEFs, along with A9, 4A, and 4AR6 cells. n = 3 per group. E) Evaluation of the mitochondrial OXPHOS function by OCR and glycolysis function by ECAR in in WT, HADHA‐HET, and HADHA‐homo MEFs. n = 3‐5. Data are mean ± SEM. *p < 0.05, ^**^P < 0.01, ns, nonsignificant. MEFs: mouse embryonic fibroblasts. WT: wildtype, HOMO: homozygous, HET: heterozygous.

As a complementary approach, we generated mouse embryonic fibroblasts (MEFs) from pregnant HADHA‐HET(HADHA±) female mice that were mated with HADHA‐HET(HADHA±) male mice.^[^ ^35^ ^]^ A total of 8 such embryos were obtained, and the genotypes of these embryos were determined as described in the methods section (**Figure** **5**A). MEFs with WT(HADHA+/+), HADHA‐HET(HADHA±) and HADHA‐KO (HADHA‐/‐) genotypes were thus generated from respective embryos. We noted that some residue HADHA protein was detected with HADHA‐/‐ MEFs, probably coming from maternal leakage. The reduction of HADHA protein in HADHA (±) MEFs was achieved at ≈37% (Figure 5B). However, such reduction didn't impact overall supercomplex assembly, while the homozygous KO significantly decreased supercomplexes assembly (Figure 5C). To further determine the role of ND6 subunit and if HADHA involves ND6 related complex I and complex I‐containing supercomplex assembly, we followed complex I assembly and further incorporation into complex I‐containing supercomplexes in both ND6 mutant and HADHA KO MEFs model systems. Samples from mitochondrial translational inhibitor chloramphenicol treatment recovery experiments were separated by BN‐PAGE, and blots were probed with antibody against NDUFA13 that recognize the early 400 KCa subcomplex I intermediate which accumulated in 4A cell resulted from the lack of ND6 (Figure 1). This 400 KDa NDUFA13 detectable subcomplex I belongs to the early assembled Pp module of the membrane arm before the step where ND6 joins the complex I assembly.^[^ ^36^ ^]^ As shown in Figure 5D, a minimum of 400 KDa was detected at the beginning of recovery in A9 cells, and it accumulated until 6 h and then decreased. In 4A cells, the 400 KDa was quite stable and even increased during the recovery. In 4AR6 cells, a residual amount of this subcomplex was detected at the beginning of the recovery, then, like in A9 cells, it accumulated until 6 h and decreased. Most interestingly, we found the appearance of this 400 kDa early assembled subcomplex I in early stage of recovery of HADHA (‐/‐) MEFs, but not in wildtype and HADHA (±) ones. Bioenergetic features were then investigated with the Seahorse XF^96^ extracellular flux analyzer on these MEFS with different levels of HADHA. As shown in Figure 5E, both basal and maximal respirations decreased significantly in HADHA (‐/‐) MEFs. In addition, electron transfer and ATP production coupling efficiency was also compromised. Meanwhile, the glycolysis was elevated with the decrease of respiration.

### 2.4 The Upregulation of HADHA during OXPHOS Stress

**Figure 6:**
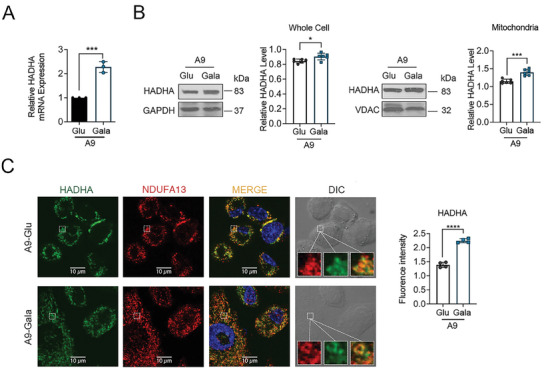
The upregulation of HADHA during metabolic shift. A) RT‐PCR analysis of the mRNA expression levels of A9‐Glu and A9‐Gala cells. A9‐Glu was regarded as control. n = 3 per group. B) Western blot of HADHA expression level in A9‐Glu and A9‐Gala cells. n = 6 per group. C) Representative images of immunofluorescence staining in A9‐Glu and A9‐Gala cells. n = 4 per group. Data are mean ± SEM. **p < 0.01, ***p < 0.001. Glu: glucose, Gala: galactose.

4AR6 was isolated when the 4A cell culture medium was switched from glucose to galactose as the major bioenergetic substrate. Under this condition, the cells were forced to rely on mitochondrial OXPHOS for ATP production.^[^ ^33^ ^]^ To test if such OXPHOS stress would impact HADHA expression, we switched the culturing media for A9 cells from regular DMEM with high glucose to galactose medium and then measured the transcription of HADHA by RT‐qPCR, followed by western blot analysis on protein levels. RT‐qPCR results showed a significant upregulation of HADHA transcription in A9 cells when the medium was switched from glucose to galactose (**Figure** **6**A). The protein levels both in whole cell and mitochondrial lysate (Figure 6B) also increased. Furthermore, enhanced expression and mitochondrial localization of HADHA in response to this OXPHOS stress was confirmed with confocal imaging (Figure 6C).

### 2.5 The Interactions Between HADHA and the OXPHOS Machinery

**Figure 7:**
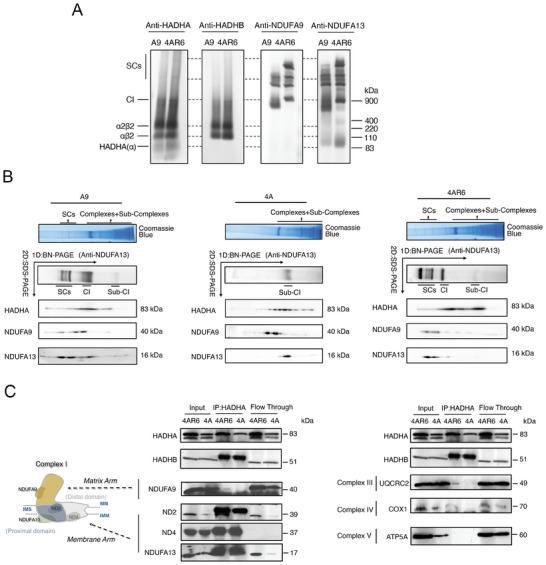
The interactions between HADHA and respiratory chain. A) Comigrations of HADHA and HADHB with respiratory complexes were shown with BN‐PAGE in A9 and 4AR6 cells. B) Associations of HADHA and respiratory complexes were detected by two‐dimensional PAGE(2D‐SDS‐PAGE) in A9, 4A, and 4AR6 cells. Coomassie blue stating and immunodetection by NDUFA13 of 1D BN‐PAGE represented the locations of respiratory complexes and SCs on the 1D BN‐PAGE. The co‐migration of HADHA with respiratory complexes and SCs was detected by immunodetection of NDUFA9 and NDUFA13 on 2D‐SDS‐PAGE. C) Direct and indirect interactions of HADHA with HADHB and representative respiratory complex subunits were demonstrated by co‐IP assay in 4AR6 and 4A cells. BN‐PAGE: Blue Native Polyacrylamide Gel Electrophoresis. IP: immunoprecipitation.

To determine if the effect of HADHA on OXPHOS assembly and function was due to a direct interaction between the OXPHOS machinery and MTP, we first analyzed the comigration of HADHA, HAHDB, and OXPHOS complexes on BN‐PAGE. As shown in **Figure** **7**A, the majority of MTP associates with the inner membrane in the form of a2b2. We also detected MTP intermediates ab2, and HADHA alone, but not core b2, probably due to the lack of stability. More interestingly, we found HADHA and HADHB co‐migrated with individual complex I and probably some supercomplex bands, particularly in 4AR6 cells (Figure 7A).

To verify that HADHA physically interacts with complex I and SCs, we performed a 2D‐SDS electrophoresis based on the 1D BNG in A9 and 4AR6 cells, with 4A cells as a negative control for complete complex I and complex I‐containing supercomplexes assemblies.^[^ ^33^ ^]^ We found HADHA indeed overlapped with complex I membrane arm subunit NDUFA13 and matrix arm subunit NDUFA9 at complex I and SCs in A9 and 4AR6 cells. Interestingly, this association with SCs was enhanced in 4AR6 cells (Figure 7B). At the same time, HADHA was also associated with complex I intermediates in A9, 4A, and 4AR6 cells, and interestingly elevated binding of HADHA with such subcomplex I was found in both 4A and 4AR6 cells (Figure 7B).

To further characterize the interaction between HADHA and the respiratory chain, an immunoprecipitation test was performed in 4AR6 cells, with 4A which forms no complete complex I and no supercomplexes as a control. Purified mitochondria of 4AR6 and 4A cells were permeabilized with digitonin and incubated with HADHA antibody. After antibody capture, the mitochondrial lysate was mixed with protein G agarose beads, and the flow through was also collected. HADHA was used to prove the success of IP experiment, and HADHB, as an established binding partner for HADHA was used as a positive control for interaction with HADHA. Consistent with the 2D gel analysis result, we found complex I membrane arm subunit ND2, ND4, and NDUFA13 precipitated readily with HADHA antibody both in 4A and 4AR6 cells (Figure 7C). However, the matrix arm subunit NDUFA9 located at the matrix arm was only pulled down by HADHA antibody in 4AR6, but not in 4A. These results again indicated that HADHA initiated the interaction with the respiratory chain at the complex I membrane arm. In addition, representative components of III (UQCRC2), and IV (COX I), but not V (ATP5A) were pulled down only in 4AR6 cells, where SCs assembly was restored in the absence of ND6 subunit (Figure 7C). These data demonstrated that HADHA binds to complex I and respiratory supercomplex.

### 2.6 Reduced HADHA Expression Decreases Respiratory Complex Activity and Enhances Steatosis in Mouse Models

**Figure 8:**
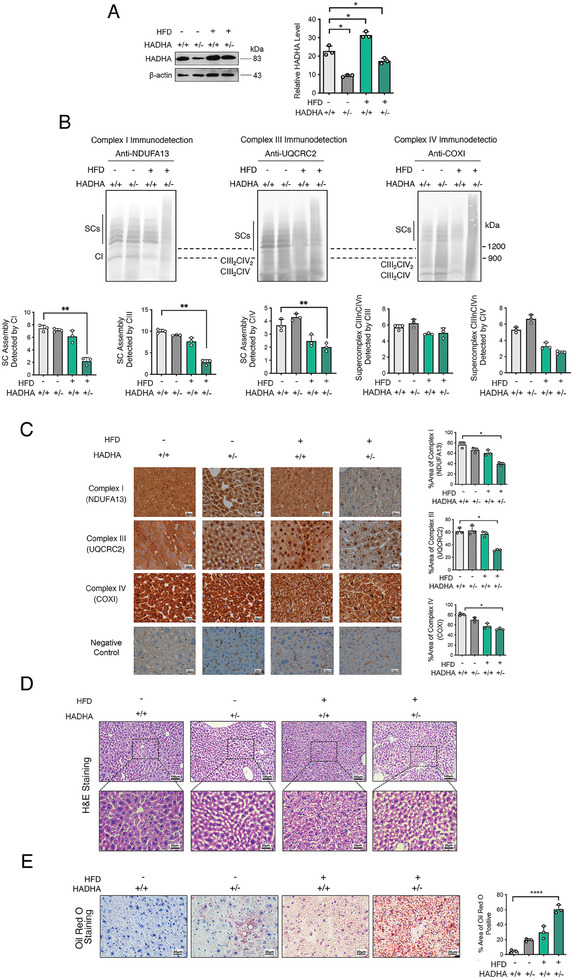
Reduced HADHA expression decreased respiratory complex activity and caused pathology in mouse liver. A) Western blot of HADHA expression level in WT‐NC, HADHA‐HET‐NC, WT‐HFD, HADHA‐HET‐HFD female mice. n = 3 per group. B) Evaluation of the complexes and supercomplexes assembly by BN‐PAGE in WT‐NC, HADHA‐HET‐NC, WT‐HFD, HADHA‐HET‐HFD. n = 3 per group. C) IHC staining in WT‐NC, HADHA‐HET‐NC, WT‐HFD, HADHA‐HET‐HFD showing the distribution and expression of HADHA and respiratory complexes. n = 3 per group. D) Representative images of hematoxylin and eosin (H&E) staining of mouse liver in WT‐NC, HADHA‐HET‐NC, WT‐HFD, HADHA‐HET‐HFD. Scale bars, 20 µm and 100µm. n = 3 per group. E) Representative images of Oil red O staining of Lipid in and quantitative analysis of lipid droplets area in WT‐NC, HADHA‐HET‐NC, WT‐HFD, HADHA‐HET‐HFD. n = 3 per group. Scale bars, 20 µm. Data are mean ± SEM.*p < 0.05; ^#^ *p* < 0.05; ***p < 0.001. WT: wildtype, HOMO: homozygous, HET: heterozygous, NC: normal chow, HFD: high fat diet, IHC: immunohistochemistry.

To explore the pathological ramifications of an FAO defect associated with HADHA deficiency and the association between FAO and OXPHOS in pathophysiological conditions, we established a mouse model with lower HADHA expression (HADHA‐HET), and then challenged it with a high‐fat diet (HFD) to the increase the stress caused by defective fatty acid metabolism. The liver is the primary organ where fatty acid oxidation occurs, and children affected by MTP deficiency usually exhibit very early hepatic dysfunction.^[^ ^37^ ^]^ To recapitulate the hepatic pathological phenotypes associated with pediatric FAO deficiency and the potential OXPHOS dysfunction, we fed 4‐month‐old HADHA‐HET mice with HFD for 2 months and then examined the liver for pathology and associated OXPHOS features. As shown in **Figure** **8**A, the level of HADHA in HADHA‐HET mice fed with normal chew (NC) was significantly reduced. Interestingly, HADHA levels were increased in the livers of WT mice fed with HFD. However, such upregulation was diminished in HADHA‐HET mice fed with HFD.

The regulation of HADHA on OXPHOS machinery in mouse liver was then determined. BN‐PAGE was performed using digitonin‐solubilized livers of mice to follow the assembly of the respiratory machinery, and we focused on supercomplex assembly based on the results obtained from the previous studies with cell lines. Overall supercomplex assemblies detected by antibodies against complexes I, III and IV were all significantly decreased in HADHA‐HET mice with HFD feeding (Figure 8B). However, interestingly, supercomplex containing only complexes III and IV, detected by either antibody against complex III or IV didn't exhibit differences (Figure 8B).

We also measured the levels of complex I, III, and IV assembly in the liver of the mouse models with the antibodies against representative subunits of complex I, III, and IV. As shown in Figure 8C, the reductions of assembly of complexes I, III, and IV were detected in HADHA‐HET mice with HFD feeding.

To determine if the decrease in supercomplex assembly will translate to the reduction of respiratory activities, we carried out enzymatic assays for the mitochondrial complex‐I and complex‐IV in the liver tissues from the mouse models. We observed that the respiratory complex activities decreased in the mice fed with HFD and the complex I activity further decreased in mice with HADHA heterozygous genotype (**Table** **1**).

**Table 1:** Measurements of NADH: Q1 and Cytochrome c activities in liver tissues from WT/NC, WT/HFD, HET/NC, and HET/HFD.

	Average nmol min^−1^ mg^−1^	
Group	NADH: CoQ1 Oxidoreductase	Cytochrome c Oxidase
WT/NC	7.720 (+1.89)	4.971 (+ 0.27)
WT/HFD	4.505 (+ 2.45)	3.980 (+ 0.8)
HET/NC	4.254 (+ 3.19)	4.653 (+ 1.26)
HET/HFD	3.444 (+ 1.50)	3.973 (+ 1.69)

The enzymatic assay was expressed in nmol/min/mg of protein. The values for NADH: Q1 Oxidoreductase and cytochrome c represent the total enzymatic activity for complex I and complex IV respectively. NADH: Q1 Oxidoreductase. 80% rotenone sensitive; and Cytochrome c Oxidase, 94% KCN sensitive. Determinations were made in triplicate. Values in parentheses are 2 SE.

Finally, to determine the impact of the decrease of HADHA and the accompanied reduction in respiratory chain function, we performed a histological analysis of liver tissues with hematoxylin and eosin (H&E) staining. As shown in Figure 8D, hepatocytes from HADHA‐HET fed with NC (HADHA ±, HFD‐) did not show pathological changes compared with the control (HADHA +/+, HFD ‐). In the HFD‐fed groups, livers obtained from WT mice (HADHA +/+, HFD+) showed microvesicular steatosis, while HADHA ±, HFD+ mice exhibited enhanced steatosis. To confirm the accumulation of the lipid, we then performed Oil Red O tests with the livers of the mice. As shown in Figure 8E, elevations of lipid droplets were found in both heterozygous HADHA mice with NC, and wild‐type mice fed with HFD. The most significant increase was recorded in heterozygous HADHA mice fed with HFD.

## 3 Discussion

FAO and OXPHOS are both critical bioenergetic pathways within mitochondria; dysfunction of either process would result in severe metabolism disorder.^[^ ^38^ ^]^ However, the underlying pathogenesis is not well understood. Previously, several FAO enzymes including the acyl‐CoA dehydrogenases (very long chain ACAD, long chain ACAD, medium chain ACAD), their redox partner ETF, and MTP have been shown to co‐migrate with OXPHOS SCs.^[^ ^24^, ^39^ ^]^ Long‐chain ACAD, was further indicated to interact directly with OXPHOS complex I at the inner mitochondria membrane.^[^ ^39^, ^40^ ^]^ The interactions between FAO and the respiratory chain have been reported in various systems. For example, HADHA was shown to be required for cardiolipin remodeling which regulates respiratory chain assembly in cardiomyocytes^[^ ^41^ ^]^; direct interaction between FAO and ETC components was also found in hearts from mouse and rat system.^[^ ^5^ ^]^ As HADHA is a primary component for FAO, and respiratory complex I is the major element of OXPHOS our study thus provides strong evidence for a coupling mechanism between these two pathways.

In this study, HADHA was initially identified due to its association with enhanced assembled and shifted SCs, under conditions where the respiratory chain included SCs assembly that was restored without an essential complex I subunit required for assembly, ND6. Direct interactions between HADHA and SCs were confirmed with biochemical and genetic characterization in 4AR6 cells. Our unique set of cell models, 4AR6 cells where SCs assembly is upregulated, together and its parental 4A cells, which have no complete complex I and thus no complex I‐containing supercomplex assembly, provide a good opportunity to investigate the supercomplex assembly. Specifically, the lack of ND6 subunit in 4A cells prevented the addition of subunits of the matrix arm to a subcomplex I which contains NDUFA13 of the membrane arm. The suppression of complex I and complex I‐containing supercomplex assembly defects observed with 4AR6 cells might happen at this stage. Thus, the interaction between FAO and OXPHOS is likely mediated by HADHA of MTP, and the complex I membrane arm of the respiratory chain. Further, the fact that HADHA was bound to subcomplex I containing the membrane arm in 4A cells, where complex I assembly fails to complete due to the lack of ND6 subunit,^[^ ^29^, ^36^ ^]^ suggests that HADHA participates in the assembly of complex I at an early stage. More importantly, the association between HADHA and respiratory chain extended in complex I and complex I‐containing supercomplexes enhanced in 4AR6 cells with the upregulation of HADHA expression, indicating HADHA facilitates the supercomplex assembly. The participation of HADHA in supercomplex assembly was also supported by a co‐IP experiment where representative subunits of complex I matrix arm, complex III and IV, but not complex V were pulled down by HADHA antibody only in 4AR6 but not 4Acells. Moreover, while HADHA association with the respiratory chain was enhanced in 4AR6 cells with upregulated overall respiratory chain assembly, in HADHA knockdown experiments, supercomplex levels were significantly decreased. Noted differences in the effects of HADHA knockdown/knockout on the respiratory chain assembly in 4AR6 cells versus MEF cells may be due to the very different metabolic, bioenergetic, and cellular contexts.

While over‐expression of HADHA was also observed in 4AR6 cells where enhanced SCs assembly and recovery of respiration capacity was achieved in the absence of complex I subunit ND6, HADHA expression was also observed to increase in cells cultured in galactose medium when metabolism is forced to OXPHOS for ATP production. However, as a consequence of OXPHOS stress, the elevated HADHA in 4A cells (even higher than 4AR6, as shown in Figure S2A (Supporting Information), didn't suppress the failure of complex I assembly in the absence of ND6 subunit. It thus explained the fact that over‐expression of HADHA alone failed to single‐handedly rescue the respiratory dysfunction in 4A cells. In these cells, the ND6 subunit is absent, and no survival colonies were obtained in galactose media when we overexpressed HADHA. Moreover, HADHA over‐expression in 4A cells (Figure S2B, Supporting Information) did not increase supercomplex assembly (Figure S2C, Supporting Information). We believe it is likely that a scenario that the enhanced binding of HADHA and CI containing was one of the consequences when 4AR6 was selected for functional complex I activity in the absence of ND6 subunit. 4AR6 cells, where the role of ND6 subunit in complex I membrane arm assembly was compensated, also likely established a particular condition where CI assembly was enhanced and the binding between MTP and OXPHOS machinery through HADHA and CI increased. The role of HADHA in complex I assembly, and likely at the stage when ND6 joined the complex was highlighted with the finding that the 400 KDa subcomplex I was also found in MEFs with homozygous knockout of HADHA.

Deficiencies in HADHA can result in LCHAD deficiency, which presents predominately as a serious liver phenotype.^[^ ^42^ ^]^ Women with heterozygous mutations in HADHA usually present with acute fatty liver failure during pregnancy.^[^ ^43^ ^]^ The HADHA‐KO mice exhibited deficiency in both MTP α‐ and β‐subunits, and these mice rapidly developed hepatic steatosis, followed by sudden death, 6–36 hours after birth.^[^ ^44^ ^]^ However, the pathological phenotypes are quite different in patients with MTP deficiency as they often carry more mild missense mutations.^[^ ^43^, ^45^ ^]^ To recapitulate the pathologic features of MTP or FAO defective patients, we created a mouse model of HADHA‐HET mice challenged with HFD feeding. Interestingly, we found that mice well‐tolerated a low HADHA level with normal chow feeding. However, with only 2 months of HFD, histopathologic changes in the liver were noticed, including increased fat deposition indicated by increasing cytoplasmic vacuoles as well as increased pyknotic nuclei, indicating enhanced steatosis. The pathological phenotypes were also correlated with the reductions in complex I‐containing respiratory SCs but not CIIICIV assembly, accompanied by decreases in respiratory complex activities in HADHA‐HET mice with HFD feeding (Figure 8B).

Mitochondrial respiratory SCs assembly and its implications in various human diseases have gained a lot of attention recently. However, the regulation of supercomplex dynamics and their physiological and pathological roles have not been well characterized. Our study has revealed a reciprocal regulation between FAO and OXPHOS. Disruption of OXPHOS machinery assembly due to the absence of complex I subunit ND6, resulted in the accumulation of lipid droplets, likely from the compromised FAO. On the other hand, interfering with FAO by knocking down HADHA disrupted the assembly of OXPHOS machinery. These results also identified the importance of a HADHA‐complex I interaction that mediates the coupling of these two vital metabolic pathways.

The coupling of OXPHOS and FAO also suggests a tissue‐specific regulation of bioenergetics. The coupling might be tighter when cells rely more on FAO for ATP production, in muscle tissue during prolonged exercise or fasting, in the liver during fasting or low carbohydrate availability, or in the heart during resting conditions. Comparatively, the coupling might be less significant in the brain where glucose is the predominant source of continuous energy fuel, in red blood cells which rely on glucose anaerobically for energy production, or in muscle tissue during bursts of intense activity.

Further exploration of this bioenergetic coupling would also promise a better understanding of pathogenesis and novel treatment for diseases involving metabolic disorders. For example, hepatic and intestinal complex I inhibition generated diabetes‐resistant phenotypes associated with alteration in both fatty acid synthesis and oxidation^[^ ^46^ ^]^; using an approved drug inhibiting MTP for other treatment purposes, also reduced tumor growth associated with a cellular redox and energy crisis.^[^ ^47^ ^]^

Altogether, our study demonstrated the FAO component HADHA was a critical factor regulating respiratory chain SCs assembly. We provided evidence for the importance of a functional OXPHOS process in FAO. A working model that accounts for these data is the FAO component MTP and the ETC are functionally linked via direct binding of HADHA to the membrane arm of complex I. This interaction is required for optimally functioning OXPHOS and FAO. Finally, our HADHA‐HET mouse model fed with HFD revealed a major contribution of OXPHOS dysfunction in the development of primary FAO disorders. OXPHOS and FAO are both widely known to play critical roles in human health. Our data show the two metabolic pathways must be considered in parallel when determining how their dysfunction leads to disease.

## 4 Experimental Section

### Cell Culture

The cell line A9 (APRT and HPRT negative derivative of Strain L) is purchased from ATCC (ATCC CCL‐1.4). It was grown in both Dulbecco's modified Eagle's medium (DMEM) supplemented with 10% fetal bovine serum (FBS) and a medium containing galactose instead of glucose. Cell line 4A, selected by rotenone‐resistance and carrying the ND6 mutation, was derived from A9 and cultivated using the same conditions as A9 with 1.2mM of rotenone.^[^ ^29^ ^]^ 4AR6 is a galactose‐resistant cell line selected from 4A and it was cultured in a medium containing galactose instead of glucose.^[^ ^31^ ^]^

### Mice and MEFs Preparation

Two pairs of HADHA‐HET(HADHA±) mice were backcrossed to the C57BL/6 inbred strain through nine generations to generate HADHA‐deficient mice and WT (HADHA+/+) littermates. The HADHA‐HOMO (HADHA‐/‐) is lethal, so only HADHA‐HET mice were used as the model for HADHA deficiency.^[^ ^51^ ^]^ The HADHA‐HET mouse genotype was determined by PCR. Four primers, named HADHA‐KO‐7352‐Common, HADHA‐KO‐6630‐Wild, HADHA‐KO‐6599‐Mutant, and HADHA‐KO‐NeoRF‐Mutant, were designed based on the genomic sequence surrounding the insertion site and the sequence of vector‐specific oligonucleotides. Primer sequences were as follows: HADHA‐KO‐7352‐Common, 5′‐CCTTCAAAGAGTTGCAACGTGTATGTG‐3′; HADHA‐KO‐6630‐Wild, 5′‐TGGGCTGTGTCAGAGCGGTGTCGTTTC‐3′; HADHA‐KO‐6599‐Mutant, 5′‐TTTTGCCAAGTTCTAATTCCATCAGAAGCT‐3′ and HADHA‐KO‐NeoRF‐Mutant, 5′‐GATAAATGCCTGTTTACTGAAG‐3′.

MEFs were obtained from 14.5‐day‐old embryos of pregnant mice from crossing HADHA‐HET male and female. WT (+/+), HADHA‐HET (±), HADHA‐HOMO (‐/‐) MEFs were determined by the genotyping method described above. MEFs were grown in DMEM supplemented with 1× non‐essential amino acids (NEAA), 1× GlutaMax (Gibco, GlutaMAX, 100×) and 10% FBS using gelatin‐coated plates.^[^ ^48^ ^]^

All the animals were maintained in a temperature‐controlled facility with a twelve‐hour light/dark cycle. Mice were randomly divided into two groups, where 20 mice were fed by normal chow (NC, D12450B, 10 kcal% fat) and 20 mice were fed by high fat diet (HFD, D12492, 60 kcal% fat) for 2 months. Mice were allowed free access to food and water. All mice used in this study were females.

All animal investigations were carried out according to the guidelines of the Animal Care and Use of UTHSCSA. The protocol was approved by the laboratory Animal Resource of UT Health San Antonio (Protocol number: 20140059AR).

Preparation of Mitochondria Fractions. Mitochondria were isolated according to previously described procedures.^[^ ^49^ ^]^ Cells were washed 3 times with ice‐cold PBS (pH 7.4), resuspended in ice‐cold RSB hypo buffer (10 mM NaCl, 1.5 mM MgCl_2_, 10 mM Tris‐HCl, pH 7.5), and transferred to a homogenizer. The cells were broken with 15–20 strokes of the B pestle. MS homogenization buffer was added to the homogenate to give a 1× final concentration. The homogenate was then centrifuged at 1300 g for 5 min at 4 °C to remove large debris and nuclei. The mitochondria were collected by centrifugation of the above supernatant at 16 000 g for 15 min at 4 °C.

### mtDNA Sequence Analysis

mtDNA sequencing was performed according to previously described procedures.^[^ ^29^ ^]^ Total DNA samples were isolated from cells with a QIAamp DNA Minikit (QIAGEN, Inc.) and then subjected to PCR amplification and sequencing with proper primers. For ND6 gene, primers ND6‐5′‐1(CACACAAACATAACCACTTTAACA) and ND6‐3′‐1 (GTAGGTCAATGAATGAGTGGTT) were sued by PCR amplification and ND6‐5′‐2 (CTTTATATCATTCCTAATTAACATC)and ND6‐3′‐2 (TGGGTGTGTTTTTCGTATGTTTG) were sued for sequencing.

Reverse‐transcriptional quantitative PCR (RT‐qPCR). Total RNA was extracted using TRI Reagent (Molecular Research Center, Inc) according to the manufacturer's protocol, and cDNA was synthesized by random hexamer and oligo(dT) primers using ThermoScript^TM^ RT‐PCR (Invitrogen). The utilized gene‐specific primers were purchased from IDT Corporation. PCR was performed for 30 cycles of 94 °C for 30 s, 55 °C for 30 s, and 72 °C for 1 min. Glyceraldehyde‐3‐phosphate dehydrogenase (GAPDH) was amplified as a control (forward, 5ʹ‐TGA AGG TCG GAG TCA ACG GAT TTG GT‐3ʹ and reverse, 5ʹ‐CAT GTG GGC CAT GAG GTC CAC CAC‐3ʹ). Specific expression of HADHA in the cell lines was established using total RNA obtained from the cells and amplified with primers for HADHA (Forward, 5ʹ‐TGC TGA CTG GCA GGA ACA TT‐3ʹ and Reverse, 5ʹ‐TGG GAC AGT CAT GGC ATA CG‐3ʹ) using SYBR^TM^ Green PCR Master Mix (ThermoFisher Scientific).

### Western Blot

The following antibodies were utilized in western blot analysis: Anti‐NDUFA13 (ab110240, Abcam), Anti‐NDUFA9 (ab14713, Abcam), Anti‐ND2 (PA5‐103952, Invitrogen), and Anti‐ND4 (PA5‐76458, Invitrogen) for complex I subunit NDUFA13, NDUFA9, ND2 and ND4. Anti‐UQCRC2 (ab1474, Abcam) for complex III subunit UQCRC2. Anti‐COX I (MABN628, Sigma) for complex IV subunit COX I. Anti‐ATP5A (ab14748, Abcam) for complex V subunit ATP5A. Anti‐HADHA antibody (ab203114, Abcam) and Anti‐HADHB antibody (sc‐271495, Santa Cruz) for mitochondrial trifunctional protein subunit‐𝛼 and ‐β. Anti‐Actin(I‐19) (sc‐1616, Santa Cruz), Anti‐GAPDH(G‐9), and antibody (MSA03) against porin (voltage‐dependent anion channel, VDAC) were used for controls. The western blot was carried out according to the protocols provided by Bio‐Rad. 30 µg of protein lysates were loaded into SDS gel. Optical densities of the immunoreactive bands were measured by ImageJ analysis software.

### Blue Native Polyacrylamide Gel Electrophoresis (BN‐PAGE)

BN‐PAGE with a 3%–11% gradient was used for separation of respiratory complexes. The digitonin: protein ratio used to solubilize OXPHOS protein from isolated mitochondria when performing BN‐PAGE was 6 g/g. 30 µg of mitochondrial protein lysates were loaded on a 1.5 × 70 × 82 mm mini gel (Bio‐Rad). The gels were run at 5 mA per gel for about 60 minutes or stopped until the dye reached ≈1/3 of the separating gel. The cathode buffer was then exchanged into colorless cathode buffer, and the gel was run until the dye reached the end of the gel. Protein complexes were detected by western blot using the antibodies described previously.^[^ ^50^ ^]^

### The Recovery Kinetics of Complexes and Supercomplexes Assembly

We cultured the cells for 8 days in the presence of 40 µg mL^−1^ chloramphenicol. Longer treatments affected cell viability. Samples were collected at different time points (0, 6, 12, 24, 48, 72, and 96 h) after chloramphenicol removal. Digitonin‐solubilized mitochondrial particles were separated by BN‐PAGE and analyzed by western blot.

### 2D BN‐PAGE/SDS‐PAGE

The mitochondrial membrane proteins of the cells were extracted and solubilized with digitonin. Samples were electrophoresed in 3–13% gradient BN gel for 4 hours and then excised corresponding lanes soaked into equilibrium buffer (containing 5% 2‐mercaptoethanol, 62.5 mM Tris‐HCL pH 6.8, 2% SDS, 10 mM glycerol) for 20mins, then run in 5–15% gradient second dimension SDS‐PAGE for 1.5 hours.

### Immunoprecipitation (IP)

IP was performed using the mitochondria IP kit (Biovision). Mitochondria Protein IP Buffer provided with the kit was added to isolated mitochondria such that the protein concentration was 1 mg mL^−1^. 1/10 volume of 10% digitonin was added to reach a final concentration of 1% detergent. 1 µL of protease inhibitor cocktail was then added to the reaction. After centrifugation at 12 000 g for 10 min at 4 °C, 30 µg of the supernatant was separated as a control fraction. The remaining supernatant was co‐immunoprecipitated in 1 ml Eppendorf tubes with 2 µg of antibodies against HADHA. The mixture was gently incubated overnight at 4 °C in a rotating shaker and centrifuged at 1000 g for 1 min to separate the flow‐through fraction. PierceTM Protein G Agarose beads (Thermo Fisher Scientific,) were added to the mixture, and the flow through was collected as a control. The beads were washed three times with 1× Wash Buffer provided with the kit. Proteins were eluted and analyzed by western blotting.

### Immunofluorescence

Immunofluorescence detection of HADHA (ab203114, Abcam), NDUFA13 (ab110240, Abcam) were performed using antigen retrieval followed by secondary detection with Alexa Fluor‐555 Goat‐Anti‐Mouse and Alexa Fluor‐488 Goat‐Anti‐Rabbit secondary antibodies (Thermo Fisher), as well as 4′, 6‐diamidino‐2‐phenylindole (DAPI) (Vector Laboratories, Burlingame, CA) for DNA. Mouse fibroblast A9 and 4AR6 were fixed by 4% paraformaldehyde and permeabilized by 200 µM digitonin. Blocking was performed with 5% Goat serum in PBS‐T (Tween 20). Microscopy was performed using a Nikon Eclipse Ti‐S microscope. Confocal microscopy was performed using an Olympus FV3000 Laser Scanning microscope. Quantification of mean fluorescence intensity was achieved using NIH FUJI Image J software. The quantitative colocalization analysis was performed using the Jacop plugin on the Fiji software. The thresholds for both Li's ICQ and Mander's coefficient were computed by the Jacop plug.

### Mass Spectrometry (MS)

Gel bands of interest were manually excised, de‐stained in 40 mM NH4CO3/50% acetonitrile, dehydrated in acetonitrile, and digested overnight at 37 °C with trypsin (Promega, sequencing grade) in 40 mM NH_4_CO_3_/10% acetonitrile. The tryptic peptides were extracted with 0.1% trifluoroacetic acid (TFA) followed by 0.1% TFA/50% acetonitrile. The combined extracts were dried by vacuum centrifugation and resuspended in 0.5% TFA for mass spectrometry analysis. The digests were analyzed by capillary HPLC‐electrospray ionization tandem mass spectrometry on an Orbitrap Velos Pro mass spectrometer (Thermo Fisher) fitted with a New Objective Digital PicoView 550 NanoESI source. On‐line HPLC separation was accomplished with an Eksigent/AB Sciex NanoLC‐Ultra 2‐D HPLC system: column, PicoFrit (New Objective; 75 µm i.d.) packed to 15 cm with C18 adsorbent (Vydac; 218MS 5 µm, 300 Å); mobile phase A, 0.5% acetic acid (HAc)/0.005% trifluoroacetic acid (TFA); mobile phase B, 90% acetonitrile/0.5% HAc/0.005% TFA; gradient 2 to 42% B in 30 min; flow rate, 0.4 µL min^−1^. MS conditions were: ESI voltage, 2.75 kV; MS1 scan range, m/z 300 – 2000; isolation window for MS/MS, 3; normalized collision energy, 30%; scan strategy, survey scan followed by acquisition of data‐dependent collision‐induced dissociation spectra of the six most intense ions in the survey scan above a threshold of 3000; dynamic exclusion, 30 s; no charge state screening; reject list of background ions (± 10 ppm). Mascot (Matrix Science, version 2.8.0.1) was used to search the spectra against a combination of the following databases: UniProtMouse downloaded on 2/8/2021 (63639 sequences; 28552995 residues); common contaminants (124 sequences; 62564 residues). Peptide tolerance was 20 ppm, and fragment tolerance, was 0.8 Da. Cysteine carbamidomethylation was set as a fixed modification and methionine oxidation, deamidation of glutamine and asparagine, and protein N‐terminal acetylation were considered as variable modifications; trypsin specified as the proteolytic enzyme, with one missed cleavage allowed. Subset searches of the identified proteins by X! Tandem, cross‐correlation with the Mascot results, and determination of protein and peptide identity probabilities were accomplished by Scaffold (Proteome Software, version 5.1.0). The thresholds for acceptance of peptide and protein assignments in Scaffold were 95% and 99%, respectively with a minimum of two peptide required, resulting in a protein‐level FDR of 0.2%.

### Bioenergetics Measurements

As previously described,^[^ ^51^ ^]^ cells were plated at a density of 10 000 cells per well in an XF^96^ cell culture microplate (Seahorse Bioscience) in 200 µL of medium and incubated overnight (14–16 h) in a humidified atmosphere of 5% (v/v) CO_2_ at 37 °C. The culture medium was changed 1 h before the assay to Seahorse assay medium supplemented with 10 mM glucose, 4 mM glutamate, and 1 mM sodium pyruvate, and then placed the plate in 37 °C incubator without CO_2_ for 1 h before the assay. The OCR and ECAR values were measured at baseline and after the sequential administration of oligomycin (2.0 µM), FCCP (1.0 µM), and rotenone (0.6 µM)/antimycin A (0.5 µM). Indices of mitochondrial function, including basal respiration, maximal respiration, and coupling efficiency were calculated accordingly.^[^ ^51^ ^]^ To determine glycolytic profiles, adherent cells were equilibrated in a glucose‐free assay medium supplemented with 2.0 mM L‐glutamine for 1 h. ECAR of the glycolytic profiles was measured at baseline and after consecutive injections of glucose (10 mM), oligomycin (1.0 µM), and 2‐DG (50 mM). We determined glycolytic profiles including glycolysis and glycolytic capacity.

To determine the exogenous fatty acid utilization, adherent cells were cultured in a growth medium. 24 h before the assay, change the growth medium to substrate‐limited medium supplemented with 0.5 mM Glucose, 1.0 mM GlutaMAX, 0.5 mM Carnitine, and 1% FBS.^[^ ^51^ ^]^ Then replace the medium with FAO Assay medium before 45 min to the assay and incubate in a non‐CO_2_ incubator for 45 min at 37 °C. Add palmitate‐BSA to the cell plate just before starting the assay, OCR of basal respiration, and maximum respiration were measured at baseline and after consecutive injections of Oligomycin (2.0 µM), FCCP (1.0 µM) and rotenone (0.6 µM)/antimycin A (0.5 µM). Data were normalized by cell numbers before the measurement and verified by protein content determined by BCA at the end, and expressed in pmol min^−1^ µg^−1^ protein for OCR values and mpH min^−1^ µg^−1^ protein for ECAR values to allow for comparisons between independent experiments. All procedures were carried out and modified according to the protocols provided by Agilent Seahorse.

### Electron Microscopy (EM)

Cells were fixed in 2.5% glutaraldehyde, 3% paraformaldehyde with 5% sucrose in 0.1 M sodium cacodylate buffer (pH 7.4), pelleted, and post‐fixed in 1% OsO4 in veronal‐acetate buffer. The cell pellet was stained overnight with 0.5% uranyl acetate in veronal‐acetate buffer (pH 6.0), then dehydrated and embedded in Embed‐812 resin. Sectioning was performed using a Leica Ultracut E microtome with a Diatome diamond knife at a thickness setting of 50 nm, with uranyl acetate and lead citrate staining. The sections were examined using a FEI Tecnai Spirit at 80 kV and photographed with an JEOL JEM‐1400 camera.

### Immunohistochemistry and H&E Staining

Organs of WT‐NC, WT‐HFD, HADHA‐HET‐NC and HADHA‐HET‐HFD mice were perfused with 4% paraformaldehyde and fixed in 10% neutral buffered formalin mixed with 70% ethanol. Immunohistochemistry and H&E staining were performed according to protocols from Cell Signaling Technology. The antibodies used for immunostaining were Anti‐HADHA (ab203114, Abcam), Anti‐Gim‐19 (ab186848, Abcam), Anti‐UQCRC2 (ab203832, Abcam), and Anti‐COX I (ab109025, Abcam). Microscopy was performed using a Nikon Eclipse Ti‐S microscope.

### Lipid Staining (Oil Red O)

The staining was done for cells as well as liver tissue sections. Briefly the A9, 4A and 4AR6 cells were grown on coverslips and incubated until the desired confluency is reached. The liver tissues were fixed in OCT solution followed by cryosectioning. The frozen tissue sections were thawed at room temperature before staining. The samples (cells and tissue sections) were fixed in 10% neutral buffered formalin followed by a quick rinse in 60% Isopropanol solution. Later the sections were stained with warmed Oil Red O stain for 15 min followed by Hematoxylin counterstain for ≈3 mins. The excess stain is washed off by dipping the section in deionized water ≈10 times. The sections were allowed to airdry and mounted using an aqueous mounting solution. The sections were imaged to see the lipid droplets at a magnification of 40X.

### Enzymatic Assays

The liver tissues were defatted and snap frozen on dry ice followed by storage at −80 °C for further use. Snap‐frozen liver samples were thawed on ice for ≈20 min. Approximately 30 mg of the tissue samples were weighed and homogenized in 600 µL of ice‐cold sucrose homogenization buffer using a glass homogenizer. The homogenate is centrifuged at 600 g for ≈15 mins at 4 °C to separate the cell debris. The supernatant was tested for protein concentration and used for further steps. For Complex 1 (CI, NADH: Ubiquinone oxidoreductase): 50 µg of protein sample was used for measuring the complex1 activity. The protein sample was added to 700 µL of distilled water and the reaction was setup in two separate tubes one tube for detecting the complex1 activity with rotenone and without rotenone. Briefly the composition of the tubes is 100 µL of potassium phosphate buffer (0.5 M, pH 7.5), 60 µL BSA free from fatty acid (50g mL^−1^), 10 µL of NADH (10 mM) and 30 µL of KCN (10 mM). To one of the tubes 10 µL of 1 mM rotenone solution is added and the mixed by inversion. The total volume of both the tubes are adjusted to 994 µL with deionized water. The reaction is initiated by the addition of 6 µL of ubiquinone (10 mM). The absorbance of the solution is measured at 340 nm for every minute. For Complex 1 V (CIV, cytochrome c oxidase) activity assay: To detect the complex IV activity 50 µg of protein sample was used. The reaction mixture is prepared by adding 60 µL of 1 mM reduced cytochrome c solution and 500 µL of potassium phosphate buffer (100 mM, pH 7.0) to 400 µL of distilled water. The volume was later adjusted to 995 µL with distilled water. The baseline reading was done at 550nm. To initiate the reaction 5 µL of sample (2.5 µg) was added and mixed by inversion and the absorbance was measured at 550 nm for every minute. To check the specificity of complex IV activity 30 µL of KCN was added (10 mM).

### Statistical Analysis

All data are presented as means ± SEM unless specified otherwise, in original or log units as appropriate.  An unpaired two‐tailed Student's t‐test was used to assess significance, except where otherwise specified. When several comparisons per data set are conducted, the Hochberg‐Benjamini FDR controlling correction for multiple testing will be applied.  If all pairwise contrasts are made, then the Tukey correction will be used.  P < 0.05 is considered significant. *p < 0.05; **p < 0.01; ***p < 0.001; ****p < 0.0001.

## Conflict of Interest

The authors declare no conflict of interest.

## Supporting information



Supporting Information

## Data Availability

The data that support the findings of this study are available from the corresponding author upon reasonable request.
