# Supplementary material for: HADHA Regulates Respiratory Complex Assembly and Couples FAO and OXPHOS
Source: Adv Sci (Weinh). 2024 Nov 3;11(47):2405147. doi: 10.1002/advs.202405147 (PMC11653673; doi:10.1002/advs.202405147)
Supplement: Supplementary file 1 — Supporting Information [file ADVS-11-2405147-s001.docx]

**
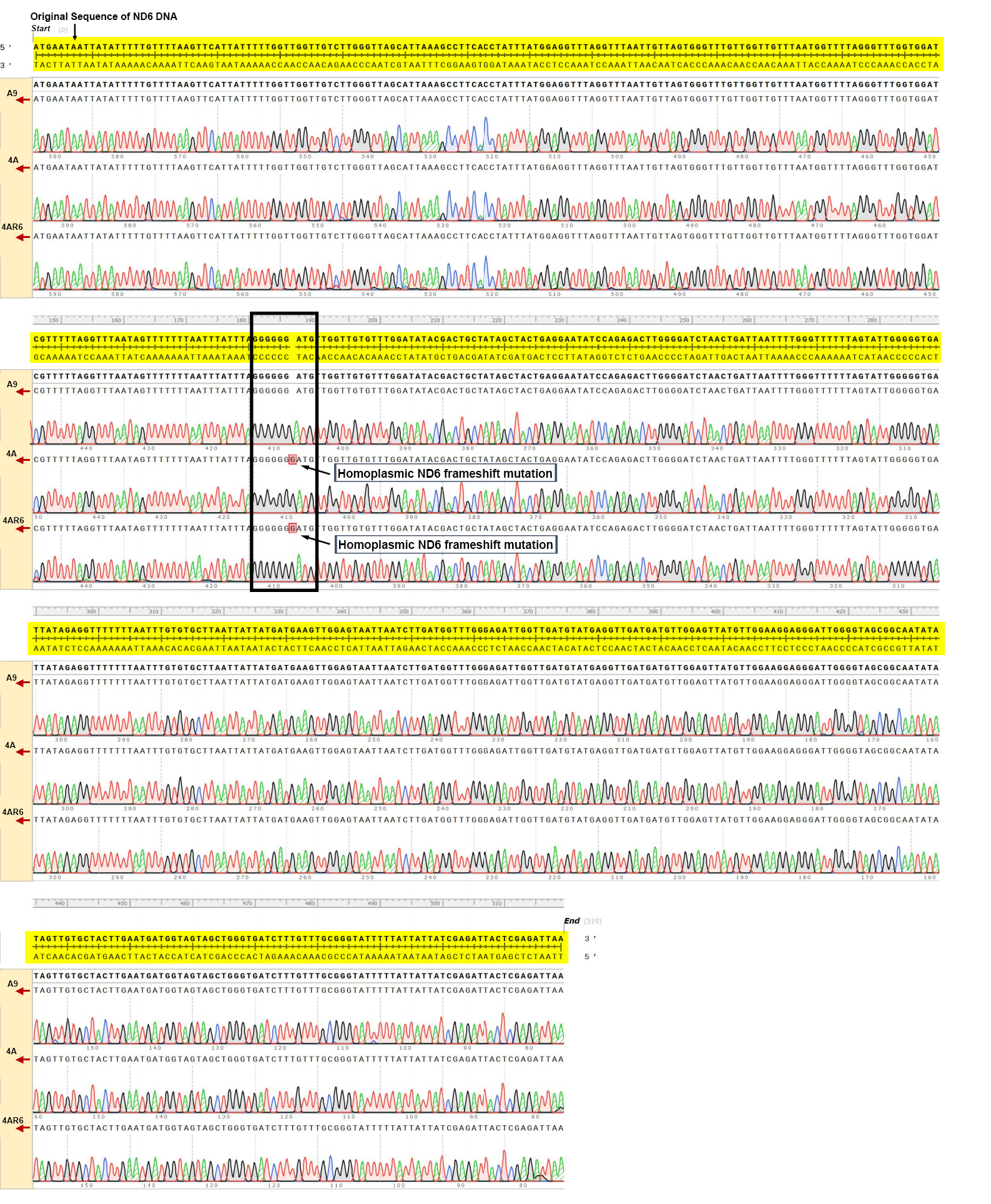
Supplementary Figure S1. ND6 Sequencing of A9, 4A and 4AR6 cells.** The standard ND6 sequences (NCBI) are highlighted in yellow, and the black rectangle marks the homoplasmic ND6 frameshift mutation in 4A and 4AR6.


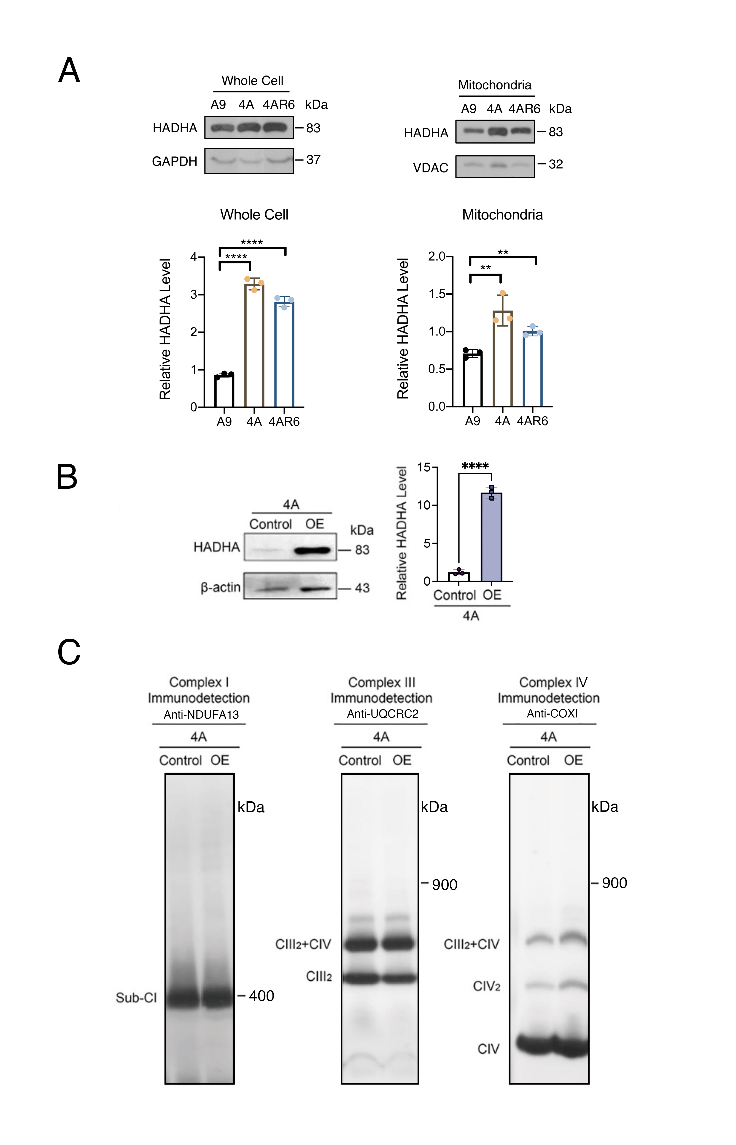


**Supplementary Figure S2. HADHA overexpression on respiratory chain assembly in mutant 4A cells. A.** Western blot of HADHA expression level in A9, 4A and 4AR6 cells. n=3 per group. **B.** Western blot of HADHA expression level in transient HADHA over-expression (OE) of 4A cells. n=3 per group. **C.** Evaluation of the respiratory chain complexes assembly by BN-PAGE in 4A-OE and 4A-control cells. n=3 per group. Data are mean ± SEM. *P < 0.001.
